# The effect of cannabinoids on wound healing: A review

**DOI:** 10.1002/hsr2.1908

**Published:** 2024-02-25

**Authors:** Aniruddha C. Parikh, Christopher S. Jeffery, Zainab Sandhu, Benjamin P. Brownlee, Lurdes Queimado, Mark M. Mims

**Affiliations:** ^1^ Departments of Otolaryngology Head and Neck Surgery The University of Oklahoma Health Sciences Center Oklahoma City Oklahoma USA; ^2^ Departments of General Surgery The University of Oklahoma Health Sciences Center Oklahoma City Oklahoma USA; ^3^ Departments of Cell Biology The University of Oklahoma Health Sciences Center Oklahoma City Oklahoma USA; ^4^ Department of Otolaryngology Head and Neck Surgery, TSET Health Promotion Research Center, Stephenson Cancer Center The University of Oklahoma Health Sciences Center Oklahoma City Oklahoma USA

**Keywords:** cannabidiol, cannabinoids, cannabis, endocannabinoid, wound healing

## Abstract

**Background and Aims:**

Cannabis and its various derivatives are commonly used for both recreational and medicinal purposes. Cannabinoids have been shown to have anti‐inflammatory properties. Inflammation is an important component of wound healing and the effect of cannabinoids on wound healing has become a recent topic of investigation. The objective of this article is to perform a comprehensive review of the literature to summarize the effects of cannabinoids on wound healing of the skin and to guide future avenues of research.

**Methods:**

A comprehensive literature review was performed to evaluate the effects of cannabinoids on cutaneous wound healing.

**Results:**

Cannabinoids appear to improve skin wound healing through a variety of mechanisms. This is supported through a variety of in vitro and animal studies. Animal studies suggest application of cannabinoids may improve the healing of postsurgical and chronic wounds. There are few human studies which evaluate the effects of cannabinoids on wound healing and many of these are case series and observational studies. They do suggest cannabinoids may have some benefit. However, definitive conclusions cannot be drawn from them.

**Conclusion:**

While further human studies are needed, topical application of cannabinoids may be a potential therapeutic option for postsurgical and chronic wounds.

## 1 INTRODUCTION

Cannabis has been cultivated throughout history since 8000 BC and has been utilized for medicine, textiles, rope, paper, and recreation. In the 19th century, it was even prescribed by physicians for gastrointestinal issues and nausea.^1^, ^2^, ^3^ However, during the 20th century, significant restrictions were implemented which limited availability for recreational use and the ability to test the medical applications of cannabis.^4^ However, since the 1990s, there has been a movement towards legalization of both medical and recreational cannabis use. With this, there has been an increase in the number of cannabis users in the United States and its use has even surpassed tobacco use with 16% of Americans reporting they currently smoke marijuana.^5^, ^6^

Today, cannabis is a term that is often used interchangeably with marijuana. However, this is an incorrect attribution. Although the precise taxonomy has not been agreed upon, the plant used in the development of marijuana is considered either a species (*Cannabis indica)* in the larger *Cannabis* genus, or a subspecies (*C. sativa* subsp. *indica*) of the *Cannabis sativa* species. The plant responsible for cannabis derived products is recognized as either the species *Cannabis sativa* in the former taxonomy or *C. sativa* subsp. *sativa* in the latter.^7^, ^8^ For the purposes of this review, we will accept the former taxonomy for simplicity's sake, recognizing the ongoing debate in this field.

Regardless of the taxonomy, a vast array of chemicals has been identified from cannabis plants. Of these, the cannabinoids have been an area of great interest. These cannabinoids include delta‐9 tetrahydrocannabinol (THC), the psychoactive compound that is predominantly found in the *C. indica* plant and is the focus of regulations, as well as cannabidiol (CBD), predominantly found in the *C. sativa* plant, which is the focus of industrial uses and nonpsychotropic medical research.^9^, ^10^

While the systemic effects of cannabis have been well studied, there is a dearth of literature addressing the effects on the wound healing process.^11^, ^12^, ^13^, ^14^ Cannabis and its components have been shown to have anti‐inflammatory properties.^15^ As inflammation is an important component within the process of wound healing, the effect of cannabis and its constituents on wound healing has become a topic of renewed investigation.^16^

The endocannabinoid system is centered around the cannabinoid 1 (CB_1_) and cannabinoid 2 (CB_2_) receptors. Traditionally, CB_1_ was reported to be found within the central nervous system while CB_2_ was located within peripheral tissues and immune cells.^17^ However, recent studies have shown this is not always the case and CB_1_ receptors may also be found peripherally.^18^ Additionally, while there are numerous natural cannabinoids which have been isolated and studied, there has been a movement to develop selective CB_1_ and CB_2_ agonists and antagonists which has furthered the understanding of how cannabinoids could play a role in cellular homeostasis.^19^, ^20^ This review describes a variety of CB_1_ and CB_2_ ligands including the CB_1_ agonist, arachidonoyl‐chloro‐ethanolamide (ACEA), the CB_2_ agonists JWH015, JWH133, and GP1a and the nonspecific CB receptor agonist WIN55,212‐2. Additionally, there are some compounds that interact with multiple receptors such as VCE‐004.8, which is a CB_2_ and PPARγ agonist. Finally, there are a variety of antagonists such as AM251 and AM630, which are CB_1_ and CB_2_ receptor antagonists, respectively (Table 1). These compounds can have varying actions on the CB receptors leading to complex downstream effects. However, the increased understanding of the roles of the CB_1_ and CB_2_ and the development of selective receptor ligands have allowed for further understanding of the effect of cannabinoids on the wound healing process.

**Table 1:** Table listing various cannabinoid agonists and antagonists.

Arachidonoyl‐chloro‐ethanolamide (ACEA)	CB_1_ agonist
JWH015	CB_2_ agonist
JWH133	CB_2_ agonist
GP1a	CB_2_ agonist
WIN55,212‐2	Nonspecific CB receptor ligand
VCE‐004.8	CB_2_ and PPARγ agonist
AM251	CB_1_ antagonist
AM630	CB_2_ antagonist

The goal of this review article is to perform a comprehensive review of the literature including in vitro, animal, and human studies, and to summarize the effects of cannabinoids on wound healing of the skin to guide potential future avenues of translational research.

## 2 METHODS

**Figure 1:**
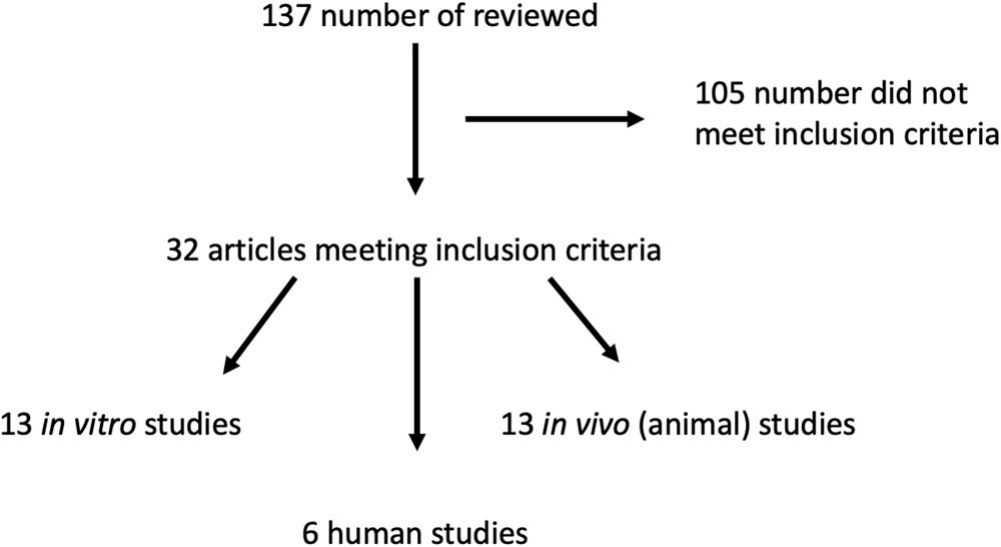
Diagram of articles reviewed.

A comprehensive review of the literature was performed using OVID Medline. Search terms included wound healing or wound inflammation and cannabis, *Cannabis sativa*, cannabidiol, cannabinoids, tetrahydrocannabinol, marijuana, hashish, or hemp. Primary basic science, animal, and human studies were identified, and abstracts were reviewed by the primary authors. Review articles and studies evaluating wound healing unrelated to the skin were excluded. Only articles in English and full text articles were included for evaluation (Figure 1). The included articles were evaluated by the authors and summarized below.

## 3 RESULTS

A total of 32 articles met the inclusion criteria. There were 13 in vitro studies, 13 in vivo studies, and six human studies (Tables 2, 3, 4). The studies are summarized below.

**Table 2:** Table summarizing the in vitro studies evaluated in this review article.

In vitro studies			
Year	Author	Cannabinoid type	Findings
2013	Ramot et al.^21^	ACEA and AM251	Keratin 6, a protein which regulates keratinocyte migration, is altered by CB_1_ agonists and antagonists
2015	Styrczewska et al.^22^	Flax fiber extract: CBD, phytosterol, and unsaturated fatty acids	Flax fiber extract showed improved wound healing, decreased inflammation, and promotion of the extracellular matrix after scratch test assays on human skin cells
2017	Bort et al.^23^	JWH015	CB_2_ agonist reduced proinflammatory markers, increased anti‐inflammatory markers, and led to faster scratch gap closure
2018	Ruhl et al.^24^	CBD	Multipotent mesenchymal stromal cells treated with CBD showed reduced oxidative stress
2019	Sangiovanni et al.^25^	CBD and *cannabis sativa* extract	Skin cells treated with these compounds showed decreased proinflammatory cytokine release and downregulation of genes which were overexpressed with TNF‐α treatment
2020	Ruhl et al.^26^	WIN55,212‐2 and JWH133	Multipotent mesenchymal stromal cells treated with a nonspecific CB receptor ligand and a CB_2_ agonist had varying effects on their differentiation which, in turn, may affect wound healing
2020	Correia‐Sa et al.^27^	N/A	Obtained skin samples from patients who underwent body contouring surgery: noted lower levels of anandamide found in patients who developed hypertrophic scars
2021	Ruhl et al.^28^	anandamide, 2‐arachidonoyl‐glycerol, and JWH133	Macrophage and mesenchymal stromal cells treated with endocannabinoids showed downregulation of their inflammatory responses
2021	Correia‐Sa et al.^29^	ACEA and AM251	Skin samples from abdominoplasty patients treated with CB_1_ agonist and antagonist noted that the agonist showed increased collagen deposition and reepithelialization
2021	Correia‐Sa et al.^29^	JWH133 and AM630	Fibroblast cells treated with a CB_2_ agonist and antagonist showed varying effects on collagen deposition depending on whether cells were treated with TGF‐β
2021	Atalay et al.^30^	CBD	Keratinocytes treated with H_2_O_2_ led to a proinflammatory state which was partially attenuated by CBD
2021	Miller et al.^31^	THC and CBD	Multiple cells lines treated with THC and CBD showed improved cell migration and specifically porcine primary fibroblasts showed faster wound closure
2021	Antezana et al.^32^	*Cannabis sativa* extract	Collagen hydrogels loaded with silver nanoparticles and *C. sativa* extract provided a matrix for tissue growth and showed improved biocompatibility compared to similar products without *C. sativa* extract

Abbreviations: TGF, transforming growth factor; TNF, tumor necrosis factor.

**Table 3:** Table summarizing the in vivo studies evaluated in this review article.

In vivo studies			
Year	Author	Cannabinoid type	Findings
2012	Zheng et al.^33^	N/A	Created wounds on mice: CB_2_ was present at baseline in uninjured tissue and its expression increased after wound injury and peaked at 3–5 days
2016	Li et al.^34^	Intraperitoneal GP1a and AM630	Mice wounds after treatment with a CB_2_ agonist showed less collagen, decreased skin thickness, and downregulation of fibrosis genes compared to an antagonist
2016	Wang et al.^35^	Intraperitoneal GP1a and AM630	Mice wounds after treatment with a CB_2_ agonist showed less wound contraction, accelerated reepithelialization, thinner dermal scar with more slender collagen fibers while the antagonist showed delayed reepithelialization
2016	Wohlman et al.^36^	N/A	Mice wounds treated with sulfur and nitrogen mustard led to upregulation of CB_1_ and CB_2_ and findings suggested CB_1_ may regulate keratinocyte proliferation whereas CB_2_ may regulate keratinocyte differentiation
2016	Del Rio et al.^37^	Intraperitoneal VCE‐004.8 or AM630	A CB_2_ and PPARγ agonist reduced collagen synthesis, deposition, and myofibroblast differentiation and reduced skin fibrosis when mice were treated with bleomycin (to create an animal model for systemic scleroderma)
2016	Mehrabani et al.^38^	Topical “New Formula” (included hemp oil)	Mice burn wounds treated with new formula showed increased wound contraction and decreased epithelialization time
2018	Du et al.^39^	Intraperitoneal JWH133, GP1a, and AM630	CB_2_ led to an anti‐inflammatory response by inhibiting proinflammatory M1 macrophages rather than increasing activity of anti‐inflammatory M2 macrophages.
2018	Klein et al.^40^	Intraperitoneal CBD	Mice wounds after CBD treatment showed lower inflammatory infiltrate on day 3. On day 7, they noted increased tissue organization and marked epithelial changes
2019	Koyama et al.^41^	Topical beta‐caryophyllene	Mice wounds treated with CB_2_ ligand showed improved reepithelialization. However, other findings suggested these may not be directly related to CB_2_ activation.
2020	Casares et al.^42^	Topical CBD	Mice skin treated with topical CBD showed increased levels of HMOX1 and wound repair keratins in mice skin
2020	McIver et al.^43^	Topical CBD	Horse wounds treated with either unique manuka factor versus topical CBD showed no difference in healing rates
2021	Ruhl et al.^24^	N/A	CB_1_ knockout mice showed delayed wound healing and CB_2_ knockout mice had increased proinflammatory cytokines but no changes to tissue regeneration
2021	Zhao et al.^44^	Topical GP1a hydrogel	GP1a hydrogel to mice wounds decreased inflammation and fibrogenesis and increased wound closure and reepithelialization

**Table 4:** Table summarizing the human studies evaluated in this review article.

Human studies			
Year	Author	Cannabinoid type	Findings
2018	Chelliah et al.^45^	Topical CBD	Case series: patients with epidermolysis bullosa had improved symptoms
2019	Palmieri et al.^46^	Topical CBD	Retrospective review: patients with various cutaneous disorders had improved symptoms
2020	Maida et al.^47^	Topical CBD, terpenes, and flavonoids	Prospective cohort study: patients with nonuremic calciphylaxis leg ulcers who failed other treatments had a good response
2021	Maida et al.^48^	Topical CBD, terpenes, and flavonoids	Prospective cohort study: patients with venous leg ulcers who failed other treatments had a good response
2021	Diaz et al.^49^	Oral THC and CBD oils	Case report: patient with pressure ulcer refractory to treatment for 5 years improved incidentally after starting CBD and THC oil
2021	Schrader et al.^50^	Topical, oral, and inhaled cannabinoids	Cross‐sectional survey: patients with epidermolysis bullosa had improvement in their symptoms

### 3.1 In vitro studies

Many studies have evaluated the effects of cannabinoids and CB receptor ligands on keratinocyte and fibroblast cells lines. Sangiovanni et al.^25^ treated keratinocyte and fibroblast cell lines with tumor necrosis factor‐α (TNF‐α) and either *C. sativa* or cannabidiol (CBD) and evaluated the effects of the proinflammatory stimulus. They noted that *C. sativa* inhibited the proinflammatory cytokine release to varying degrees whereas CBD showed some inhibition but not to the same degree as *C. sativa*. Finally, they showed that both compounds were able to downregulate genes which were overexpressed after TNF‐α treatment.^25^ Miller et al.^31^ treated bone marrow‐derived stem cells, adipose‐derived stem cells, and porcine primary fibroblasts with THC and CBD and noted improved cell migration. Additionally, they found when porcine primary fibroblasts where exposed to CBD, there was a 75% faster wound closure.^31^ Ramot et al.^21^ found the expression of keratin 6, a protein which downregulates keratinocyte migration, decreased when keratinocytes were treated with arachidonoyl‐chloro‐ethanolamide (ACEA), a CB_1_ agonist, and increased when keratinocytes were treated with a CB_1_ antagonist. They hypothesized the effects of CB_1_ stimulation on keratin 6 could be exploited to modulate reepithelialization in wound healing.^21^ Bort et al.^23^ showed that JWH015, a CB_2_ agonist, reduced proinflammatory cytokines and increased anti‐inflammatory compounds, such as transforming growth factor‐β (TGF‐β), in keratinocytes and fibroblasts treated with lipopolysaccharide, and induced a faster gap closure during a scratch test assay. The cells were treated with CB_1_ and CB_2_ antagonists and they noted these effects were likely modulated through both receptors. Additionally, they utilized porcine skin as an ex vivo skin model and found that JWH015 had sustained and low transdermal distribution which may have promising therapeutic potential.^23^

Ruhl et al.^24^ treated cells with lipopolysaccharides and observed that co‐treatment with CBD reduced the oxidative stress on cells. Lipopolysaccharides also inhibited further cell differentiation, and this was attenuated by CBD.^24^ They then evaluated the effects of WIN55,212‐2, a nonspecific CB receptor ligand, and JWH133, a CB₂ agonist, on multipotent mesenchymal stromal cells from subcutaneous adipose tissue and noted these compounds had varying effects on their differentiation. Interestingly, they also noted that both WIN55,212‐2 and JWH133 increased hepatocyte growth factor which is known to stimulate migration and proliferation of keratinocytes and is important in cutaneous wound healing.^26^ They then treated macrophages and mesenchymal stromal cells with lipopolysaccharides and evaluated the inflammatory response after treatment with the endocannabinoids anandamide, 2‐arachidonoyl‐glycerol, and JWH133 and noted they decreased the inflammatory response of M1 macrophages. This decrease was less significant in activated mesenchymal stromal cells.^28^

Correia‐Sa et al.^27^ collected skin samples from patients who underwent body contouring surgery and noted lower levels of anandamide were found in hypertrophic scars compared to normal scars. They suggested that reduced anandamide is potentially related to increased inflammation or a prolonged inflammatory phase which predisposed patients to scar hypertrophy.^27^ They then collected skin samples from abdominoplasty patients and treated cells with ACEA and AM251, a CB_1_ antagonist. They noted increased collagen deposition with ACEA and decreased collagen deposition with AM251. Additionally, they performed ex vivo reepithelization studies and noted ACEA speeds reepithelialization.^29^ This same group then evaluated the effects of TGF‐β on fibroblast cells and treated these cells with JWH133 and AM630, a CB_2_ antagonist. They noted TGF‐β increased α‐SMA expression, which is a marker for myofibroblast differentiation, and increased expression of CB_2_. Both JWH133 and AM630 led to decreased collagen deposition and α‐SMA expression after exposure to TGF‐β. When the fibroblasts were not treated with TGF‐β, only AM630 led to decreased collagen deposition and α‐SMA expression. This study showed the complex interplay of CB_2_ on the wound healing process.^51^

Styrczewska et al.^22^ performed scratch test assays with hydrophobic flax fiber extract, which contained CBD, and demonstrated improved wound healing via inhibition of chronic inflammation and promotion of extracellular matrix remodeling and skin cell migration. The authors suggested the improvement is secondary to two components: presence of CBD and the content of phytosterol. Atalay et al.^30^ treated keratinocytes with hydrogen peroxide which led to a proinflammatory state which was partially attenuated by CBD.

Some authors also evaluated how CBD may assist in modulating wound healing dressings. Antezana et al.^32^ developed collagen hydrogels loaded with silver nanoparticles and *C. sativa* extract. They found this compound reduced bacterial growth and provided a matrix that allowed for tissue growth. The silver nanoparticles and collagen gels were shown to be toxic to human cell lines; however, with the addition of *C. sativa* there was improved biocompatibility due to decreased cell toxicity and continued antimicrobial activity.^32^

### 3.2 Animal studies

Ruhl et al.^52^ evaluated CB_1_ and CB_2_ knockout mice by creating a wound which was analyzed over a 2‐week period. They found CB_2_ knockout mice had increased proinflammatory cytokines but no changes in tissue regeneration. CB_1_ knockout mice showed delayed wound closure early in the healing process suggesting that while previously not considered as present within the immune system, CB_1_ does play a role in modulating inflammation during wound healing.^52^

Zheng et al.^33^ created a 1.5 cm incision on mice and evaluated these wounds at variable time intervals for the for the timing of CB_2_ expression. They found that while CB_2_ was present at baseline within uninjured tissue, its expression within inflammatory cells significantly increased after wound injury. They found macrophages and myofibroblasts with CB_2_ reached their maximum numbers at 3 and 5 days, respectively.^33^ They then evaluated the effects of CB_2_ activation on fibrogenesis. They created two 6 mm wounds on mice dorsum and subsequently treated the mice with GP1a, a CB_2_ agonist, AM630, and a control, intraperitoneally. They found mice treated with GP1a had less collagen deposition, more slender collagen fibers, and decreased skin thickness whereas those treated with AM630 showed opposing results. Additionally, the GP1a group showed decreased expression of cytokines involved in fibrogenesis and downregulation of fibrosis associated genes whereas the AM630 group showed increased levels of these cytokines and upregulation of these genes.^34^ In another study, they evaluated the effects of GP1a and AM630 on wound inflammation and reepithelialization after wounds were created on mice. When treated with AM630, there was delayed reepithelization. When treated with the GP1a, they noted decreased wound contraction, accelerated reepithelialization, thinner dermal scar with more slender collagen fibers, increased interferon‐γ, and decreased epidermal hypertrophy, granulation tissue, inflammatory infiltration, and expression of inflammatory proteins. Additionally, there was more epithelial to mesenchymal transition with increased ability for cell migration. Overall, these findings suggest CB_2_ receptor activation attenuates wound inflammation and fibrogenesis.^35^ In 2018, they created cutaneous wounds on mice and treated them with JWH133, GP1a, and AM630 to evaluate the specific macrophage response noted in the wound bed. They noted that while both M1 and M2 macrophages were present within the wound bed after injury, M1 macrophages were seen earlier in the injury process whereas M2 macrophages were more present in the later stages. They also found the presence of M1 macrophages was likely related to expression of the CB_2_ and suggested its activation led to an anti‐inflammatory response by inhibiting the proinflammatory M1 macrophages rather than increasing the activity of the anti‐inflammatory M2 macrophages.^39^ Overall, the studies from this group describe the critical role that CB_2_ activation plays in improved wound healing.

Klein et al.^40^ created oral tongue ulcers in rats and treated them with varying concentrations of CBD and a control, intraperitoneally. They grossly and pathologically analyzed the wound at days 3 and 7. They noted no gross difference between the groups. However, they found that at day 3 the groups treated with CBD had a significantly lower level of inflammatory infiltrate compared to the vehicle groups, but this change was not noted on day 7. Interestingly, they noted increased tissue organization at day 7 and more marked epithelial changes in the CBD group suggesting its favorable effects on reepithelization.^40^

Zhao et al.^44^ created a hydrogel containing Gp1a and administered this locally to 4 mm punch wounds created on mice. They found after treatment with the Gp1a hydrogel, there was increased levels of CB_2_ expression 4 and 8 days after surgery suggesting the hydrogel allowed for gradual release of Gp1a over 8 days. Additionally, the Gp1a hydrogel group showed decreased messenger RNA (mRNA) expression of inflammatory cytokines, fibrosis, skin thickness, and mRNA and protein levels of collagen I compared to the control. Finally, the Gp1a hydrogel group showed faster wound healing, longer epithelial sheets, and increased levels of protein markers characteristic of epithelial‐mesenchymal transition.^44^

Koyama et al.^41^ treated mice wounds with beta‐caryophyllene, a compound present in herbs and spices and a ligand of CB_2_. They noted improved reepithelialization, increased markers of reepithelization, and increased cell proliferation throughout the wound bed. However, while some of their experiments suggested these changes may be related to CB_2_ activation, others suggested they may not be directly related to CB_2_ activation given that CB_2_ was downregulated in the beta‐caryophyllene group.^41^ Wohlman et al.^36^ evaluated the effects of sulfur mustard and nitrogen mustard on mice. Administration of the mustard compounds led to epidermal hyperplasia and upregulation of CB_1_, CB_2_, PPARα, and fatty acid amid hydrolase (FAAH). They noted there was a significant increase in these proteins immediately after exposure to the vesicants, and the increase in CB_1_, CB_2_, and PPARα was persistent in the hyperplastic epidermis. Their results suggested CB_1_ may be important in regulating keratinocyte proliferation given it was upregulated in proliferating cells whereas CB_2_ may regulate keratinocyte differentiation as it was upregulated within sebaceous glands.^36^ Del Rio^37^ developed a CB_2_ and PPARγ agonist, VCE‐004.8. They showed TGF‐β induced collagen synthesis, deposition, and myofibroblast differentiation were decreased when cells were pretreated with VCE‐004.8 and subsequently stimulated with TGF‐β. Interestingly, scratch test assays showed that pretreatment with VCE‐004.8 attenuated wound closure induced by TGF‐β, further emphasizing the complex interplay of cannabinoid receptors and wound healing. To create an animal model of systemic scleroderma, they treated mice with bleomycin injections which increased dermal thickness and collagen content and they showed treatment with VCE‐004.8 reduced skin fibrosis. Whereas when pretreated with a CB_2_ or PPARγ antagonist before treatment with VCE‐004.8, a reduced antifibrotic response was noted, suggesting the observed effect is dependent of both PPARγ and CB_2_.^37^ Casares et al.^42^ evaluated the effects of CBD on HMOX1 which has several important antioxidant and anti‐inflammatory properties. When keratinocyte cells were treated with CBD, there was noted to be increased HMOX1 which was likely secondary to BACH1 degradation. They also performed in vivo studies on mice using topical CBD which showed increased levels of HMOX1 and wound repair keratins in mice skin.^42^

Mehrabani et al.^38^ described the beneficial effects of “new formula” (NF), a combination of sesame, wild pistachio, hemp, and walnut oil, on burn wounds in mice. They treated the burns with either nothing, NF, or silver sulfadiazine and noted the NF group had significantly increased wound contraction and decreased epithelialization time compared to the other groups.^38^ McIver et al.^43^ treated open wounds created on limbs of horses with either unique manuka factor (UMF) 5 alone (which has minimal antimicrobial activity), UMF 20 manuka honey (which has superior antimicrobial activity), saline, or UMF 5 mixed with 1% CBD. They found no difference in healing rates. They postulated that since all horses had at least one wound treated with CBD, the CBD had a systemic anti‐inflammatory effect leading to similar would healing times; however, no further testing was conducted to validate this hypothesis.^43^

### 3.3 Human studies

There were limited studies which examined the effects of cannabinoids on humans and wound healing. The articles identified were mainly observational studies and case reports.

Diaz et al.^49^ published a case report where they described a patient who was treated with oral cannabis oil, which included a combination of CBD and THC, to treat a chronic pressure ulcer. Specifically, the patient was administered a CBD‐dominant oil once daily and two different THC‐dominant oils twice daily. They noted significant improvement in the healing of the pressure ulcer at 2 weeks and almost complete closure by 2 months. A foam padded dressing was the only other intervention applied which may have confounded the effect of the cannabis oil treatment.^49^ Maida et al.^47^, ^48^ discussed topical cannabis‐based medicines (TCBMs) which had varying amounts of THC, THCA (delta‐9 tetrahydrocannabinolic acid), and CBD. They have published two case series describing the treatment of venous stasis ulcers and nonuremic calciphylaxis ulcers with topical TCBMs. In the venous leg ulcer study, they treated 14 patients with 16 recalcitrant leg ulcers with TCBMs and compression bandaging. They reported complete closure in 11 patient and 13 wounds within a median of 34 days. In the nonuremic calciphylaxis leg ulcer study, two patients with multiple recalcitrant ulcers were treated with TCBMs. Both patients had complete closure of their wounds within a mean of 76.3 days. Although they reported a possible benefit in both studies, their study design did not include a control group making it difficult to determine if the TCBMs led to the wound closure.^47^, ^48^

In another case series, three patients self‐initiated various topical formulations of CBD to treat epidermolysis bullosa (EB) lesions. The formulations were not consistent among the cases nor was the application standardized. The patients were being treated with a variety of ineffective wound care measures, and after starting topical CBD, they reported significant improvement of pain, with reduction of opioid use as an endpoint, and concluded there was acceleration in wound healing. However, this was anecdotal and not based on objective measures.^45^ In an international cross‐sectional study by Schraeder et al.,^50^ a survey was sent out to EB patients and their caretakers. Seventy‐one respondents reported utilizing both topical and oral CBD in varying concentrations and in conjunction with other remedies. Pain and pruritus were self‐reported to decrease by 3 points on a numerical rating scale from 0 to 10 (*p* < 0.001 for both) after CBD use. Most respondents reported CBD use improved their overall EB symptoms (95%), pain (94%), pruritus (91%), wound healing (81%), and decreased the use of pain medication (79%).^50^ Palmieri et al.^46^ retrospectively evaluated the effects of CBD ointment administered to patients 20 patients with moderate to severe psoriasis (five patients), atopic dermatitis (five patents), and the resulting scars from these disorders (10 patients). They were treated with an organic skin care ointment which contained CBD oil twice daily for 3 months. They objectively measured skin hydration, transepidermal water loss, and skin elasticity and noted improvements in all parameters. Additionally, they reported improvement in cutaneous blemishes and scars and fewer pustules and papules. Finally, they noted significant improvement in patient's Psoriasis Area Severity Index scores.^46^

## 4 DISCUSSION

From some of the earliest recorded history, the medicinal use of cannabis and its derivatives has been an area of keen interest. Although many historical reports exist to profess claims of the capabilities of these compounds, only more recently has the scientific process been applied to determine what, if any, role they may have. Here, we reviewed the available literature on cannabis and its derivatives as they relate to cutaneous wound healing.

In vitro studies have offered a variety of targets in which cannabis containing compounds may influence wound healing. Overall, these studies suggest a positive effect on wound healing by cannabinoids. One study even utilized human skin samples and noted hypertrophic scars had lower levels of anandamide when compared to those with normal scars.^27^ Perhaps the most compelling of target based on these studies is CB_2_ and its modulation of the acute phase inflammatory response. Later animal studies offered a potential explanation for this observation through the decreased expression of M1 macrophages in early wound healing when the wound was exposed to CB_2_ agonists.^39^ Several subsequent studies have corroborated the beneficial effects seen with CB_2_ agonists in wound healing. In addition to its effect on early inflammatory markers, CB_2_ agonists appear to improve wound reepithelialization as well as lead to a decrease in fibrosis and epidermal hypertrophy.^29^, ^34^, ^35^, ^37^, ^38^ Although the available human studies addressing cannabinoids and skin wound healing have promising results, the relative paucity of more robust study methods (e.g., randomized control trials, prospective case‐control studies, etc.) limits their applicability. Nonetheless, these studies serve as valuable proof of concept reports that future, more robust, studies may build upon.

In addition to discerning the physiologic method of action of cannabinoids and wound healing, another important avenue for research lies in medication delivery. Many of the animal studies reviewed here utilized intraperitoneal injection to deliver the experimental drug, even though systemic application may not be the ideal delivery mechanism to examine more localized effects. These systemic administrations increase technical difficulty as well as introduce unnecessary side effects for subjects. Topical and local application can be performed by several methods including oils, ointment, paste, local injection after wound closure, or impregnation into dressings. Additionally, some formulations may allow for sustained drug delivery. For example, Zhao et al.^44^ developed a hydrogel which required only one application. If further human studies do show improved wound healing after cannabinoid application, optimizing drug delivery methods will be critical to improved outcomes.

As cannabis and cannabinoid‐related products become more pervasive in the lives of patients, it is important for researchers and clinicians alike to recognize this movement. Although the exact effect these compounds have on various conditions remains to be elucidated, their use should be recognized and documented in detail. It is no longer sufficient to only screen for recreational marijuana use. There is now a growing body of literature that demonstrates these legal over the counter and prescription compounds have active local and systemic effects that may help, or interfere with, conditions and treatments experienced by patients. Thus, further efforts must be made to accurately document the type and amount of cannabinoids a patient is utilizing.

When discussing new medical treatments, side effects must be considered. The negative systemic effects of cannabinoids are well known and include exacerbations of various psychiatric conditions and both acute and long‐term cognitive deficits. They have been shown to affect the cardiovascular system and may be associated with myocardial infarction, cardiomyopathy, and sudden cardiac death. When smoked, they can have deleterious effects on the respiratory system. Additionally, they can negatively affect the reproductive system.^53^ Topical cannabinoid treatment will likely prevent many of the systemic side effects by directing the treatment directly to the tissue of interest. However, topical medications have a risk of acute skin reactions such as contact dermatitis or urticaria and this would also be a concern for topical cannabinoids. Interestingly, the endocannabinoid system may attenuate contact dermatitis and play a protective in these reactions.^54^ A recent study evaluated the dermatological side effects of topical cannabinoids, including CBD and hemp seed oil. Overall, they found no significant reactions via patch testing and only saw a mild phototoxicity at 48 h when treated with hemp seed oil. The findings suggest that these products appear to be safe. However, given the various formulations that are in the market and the difficulty in regulating topical cannabinoids, these results are likely not generalizable to all cannabinoid products.^55^ Further research is needed to understand the full spectrum of side effects of topical cannabinoids.

## 5 CONCLUSION

Cannabinoids appear to improve cutaneous wound healing through a variety of mechanisms, most notably through the CB_2_ receptor. Additional, more robust, in vivo and human studies are needed to better define these mechanisms as well as examine cannabinoid's role in human cutaneous wound healing. Finally, while the risks of systemic cannabinoids are well described, the risks of topical cannabinoids are not well known. Although their side effects do appear to be relatively mild, further studies are needed to understand the adverse effects of topical cannabinoids.

All authors discussed the results, reviewed the manuscript, and approved the final submitted version. Each author contributed important intellectual content during manuscript drafting or revision, agrees to be personally accountable for the author's own contributions, and to ensure that questions pertaining to the accuracy or integrity of any part of the work are appropriately investigated and resolved.

## AUTHOR CONTRIBUTIONS

**Aniruddha C. Parikh**: Conceptualization; data curation; investigation; methodology; writing—original draft; writing—review and editing. **Christopher S. Jeffery**: Conceptualization; data curation; investigation; methodology; writing—original draft; writing—review and editing. **Zainab Sandhu**: Investigation; writing—original draft; writing—review and editing. **Benjamin P. Brownlee**: Investigation; writing—original draft; writing—review and editing. **Lurdes Queimado**: Conceptualization; investigation; methodology; supervision; writing—review and editing. **Mark M. Mims**: Conceptualization; investigation; methodology; supervision; writing—review and editing.

## CONFLICT OF INTEREST STATEMENT

The authors declare no conflicts of interest.

## TRANSPARENCY STATEMENT

The lead author Mark M. Mims affirms that this manuscript is an honest, accurate, and transparent account of the study being reported; that no important aspects of the study have been omitted; and that any discrepancies from the study as planned (and, if relevant, registered) have been explained.

## Data Availability

Data sharing is not applicable to this article as no new datasets were generated or analyzed during the current study.
